# Effects of circadian misalignment on sleep in mice

**DOI:** 10.1038/s41598-018-33480-1

**Published:** 2018-10-26

**Authors:** Sibah Hasan, Russell G. Foster, Vladyslav V. Vyazovskiy, Stuart N. Peirson

**Affiliations:** 1 Sleep and Circadian Neuroscience Institute, Nuffield Department of Clinical Neurosciences, University of Oxford, Oxford Molecular Pathology Institute, Dunn School of Pathology, South Parks Road, Oxford, OX13RE United Kingdom; 2 Department of Physiology, Anatomy and Genetics, University of Oxford, Parks Road, Oxford, OX1 3PT United Kingdom

## Abstract

Circadian rhythms and sleep-wake history determine sleep duration and intensity, and influence subsequent waking. Previous studies have shown that T cycles - light-dark (LD) cycles differing from 24 h - lead to acute changes in the daily amount and distribution of waking and sleep. However, little is known about the long-term effects of T cycles. Here we performed continuous 10 day recording of electroencephalography (EEG), locomotor activity and core body temperature in C57BL/6 mice under a T20 cycle, to investigate spontaneous sleep and waking at baseline compared with when the circadian clock was misaligned and then re-aligned with respect to the external LD cycle. We found that the rhythmic distribution of sleep was abolished during misalignment, while the time course of EEG slow wave activity (1–4 Hz) was inverted compared to baseline. Although the typical light-dark distribution of NREM sleep was re-instated when animals were re-aligned, slow wave activity during NREM sleep showed an atypical increase in the dark phase, suggesting a long-term effect of T cycles on sleep intensity. Our data show that circadian misalignment results in previously uncharacterised long-term effects on sleep, which may have important consequences for behaviour.

## Introduction

Circadian rhythms are endogenously generated oscillations with a period ~24 h that persist in the absence of environmental time cues^1^. Such rhythms are a fundamental property of virtually all organisms, and have evolved as an adaptation to predictable environmental changes associated with the solar cycle. In mammals, the central circadian pacemaker located within the hypothalamic suprachiasmatic nuclei (SCN) drive circadian rhythms of physiology and behaviour throughout the body. The circadian system is organized in a hierarchical manner with the SCN driving physiological rhythms such as locomotor activity and core body temperature, as well as coordinating peripheral oscillators via neural, endocrine and behavioural signals^2,3^.

Disruption of the circadian system is associated with negative health outcomes, such as those observed in shift work, circadian rhythm sleep disorders and insomnia^4–8^. Altered light-dark (LD) cycles have been widely used in animal models to study the effects of circadian disturbances on physiology and behaviour^9–13^. The capacity to entrain to an LD cycle differing from 24 h is commonly investigated using T-cycle paradigms. When the period of the LD cycle is close to the period of the biological clock, entrainment is still possible. However, more extreme T-cycles (e.g. <20 h or >28 h) prevent entrainment, resulting in animals displaying a period of around 24 h despite the prevailing LD conditions^14–16^. In CD1 mice, the effects of a range of different light-dark cycles (between 21–27-h) on locomotor activity rhythms have been investigated^9^. Mice were able to entrain to these new cycles, with the exception of very short T-cycles (T21 and T22), when animals showed a dissociation of the activity rhythm into two components - one corresponding to the period of the LD cycle, the second free-running with a period around 24h^14^. In rats the majority of animals under a T22 LD cycle similarly expressed two periodic components in both locomotor activity and core body temperature^11^. Subsequent studies using the same T22 paradigm in rats were able to dissociate the circadian regulation from the light inhibition of melatonin release^16^. Studies have also used T cycles to investigate the effects of circadian misalignment in healthy CD1 mice^12^, as well as in a mouse model of cardiac hypertrophy^13^. It was found that under T20 cycles C57BL/6J (B6) mice showed an accelerated weight gain, accompanied by changes in leptin and insulin, altered morphology of neurons in the medial prefrontal cortex, and behavioural impairments^12^. In a cardiac hypertrophy model, T20 cycles exacerbated disease pathophysiology^13^. Finally, studies in B6/129 F1 hybrid mice housed under T7 cycles have shown that animals show depression-like behaviours with impaired hippocampal long-term potentiation and learning^17^.

Sleep is a complex biological process arising from the interaction of numerous brain regions and neurotransmitter systems^18–20^. Sleep is regulated by two processes - the circadian Process C, that varies over 24 h, and a homeostatic Process S that increases during wakefulness and decreases during sleep^21–24^. Growing evidence has suggested a continuous interaction between C and S rather than interaction only at discrete time-points^25^. Changes that occur over the sleep-wake cycle as well as following sleep deprivation have important consequences for mood^26^, synaptic plasticity and cognition^27–29^. However, only relatively few studies in animals have addressed the effect of circadian misalignment on sleep^10,11,17,30^. Recently, circadian effects on NREM sleep spectral EEG characteristics have been investigated in rats using a constant ‘sleep pressure protocol’, which revealed that activity in the 7–25 Hz frequencies in NREM sleep EEG are influenced by circadian regulation while Slow Wave Activity (1–4 Hz, SWA) is more influenced by preceding sleep-wake history^31^. A follow up study showed that high-frequency EEG activities in REM sleep (>19 Hz) and waking (>10 Hz) are sleep-wake dependent and may be under control of the sleep homeostatic mechanism, whereas activity in the 3–7 Hz frequencies in the REM sleep EEG are under strong circadian regulation^32^. By contrast, slow theta activity in waking (5–7 Hz) were influenced by circadian processes as well as locomotor activity and exploratory behaviour.

Despite the use of T-cycles to disrupt circadian rhythms, studies on the effects of these protocols on sleep are limited. This has important consequences when considering the effects of circadian perturbations on cognitive and emotional behaviour, which are known to be affected by sleep disruption. Recent studies have shown that short T-cycles cause a significant increase in EEG slow wave activity during NREM sleep, and a loss of the periodic changes in the power of theta (5.5–10 Hz) and gamma (35–50 Hz) frequencies during waking^33^. However, under such conditions, sleep may dynamically change from day-to-day, depending upon the relationship between internal biological time and the external environment. We hypothesised that the effects of T-cycles on sleep would be greatest when internal and external time were misaligned. Furthermore, we predicted that specific effects on EEG would persist even when internal and external time were realigned (when the ~24 h component would be approximately back in phase with baseline after seven 20 h cycles). To investigate these effects, here we assessed the effects of T-cycles on sleep and waking in C57BL/6J mice using a T20 cycle. We show that misalignment of internal and external time results in changes in amount and relative distribution of vigilance states. Moreover, we show that even when approximately re-aligned, despite the amount and distribution of sleep appearing comparable, mice still show changes in EEG spectra that may have implications for subsequent behaviour.

## Results

### Circadian rhythms of locomotor activity and core body temperature under T20 cycles

**Figure 1:**
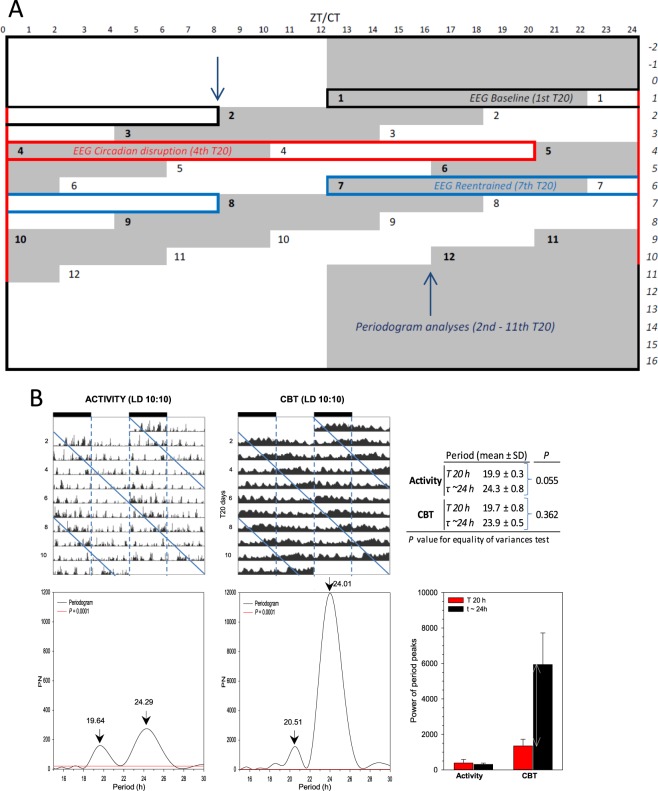
(**A**) Raster plot of the short day (20-h) forced desynchrony protocol in mice (n = 5). The first 10:10LD (T20) cycle (baseline/aligned) started at dark-onset (ZT12 of the previous 12:12LD). The dark-onset of the fourth T20 (misaligned) occurred ‘out of the phase’ (beginning of the subjective day based on preceding T24). The dark-onset of the seventh T20 (re-aligned) occurred ‘back in phase’ (beginning of the subjective night based on preceding T24). Periodogram (Lomb-Scargle; *P* < 0.0001) analyses were performed on the activity/temperature data from 2^nd^ T20 cycle until 11^th^ T20 cycle (between dark blue arrows). EEG data were scored for vigilance states (VS) of the baseline (black rectangular open box), misaligned (red box) and re-aligned (blue box) cycles. (**B**) Actograms revealed both a 20 and a 24-h rhythm component for gross activity and core body temperature under T20 conditions. (*Upper*) Double plotted actograms for motor activity and temperature of a representative mouse under a 20-h LD cycle. The vertical dashed lines of the actograms denote the light and dark phases of the 20-h rhythm component. The diagonal lines of the actograms indicate the onset of the 24-h rhythm component. (*Lower*) Lomb-Scargle periodograms of the time series represented on the actograms. The analysis yielded statistically significant peaks (*P* < 0.0001) for the *τ ~24*-h and the *T 20*-h rhythms (black arrows). The numbers on top indicate the period of the significant peaks in hours. (*Top-right*) Table representing the averaged periods (AVG ± SD, B6: *n* = 5) of the 20 and 24-h rhythms. Statistics are indicated by *P* values for the equality of variance test showing that the 20-h component of activity (19.9-h) had a smaller variance (SD = 0.3) compared to the 24-h component of activity. (*Bottom-right*) Averaged histograms (AVG ± SEM) of the power of period peaks revealed a trend for an increase in the *τ ~24*-h core body temperature power compared to *T 20*-h core body temperature (indicated by the double-headed arrow; *P* = 0.062, paired *t-*test).

C57BL/6 (B6) adult mice were housed under a T-cycle of 20 h (10:10 Light-Dark) for 11 days (Fig. 1A). Individual actograms were plotted for both locomotor activity and core body temperature, and revealed both a 20 and a 24-h periodic component for both parameters in all mice (Fig. 1B). The 24 h period observed is slightly longer than the usual free-running period in C57BL/6J mice, suggesting that this non-entrained period may be the result of repeated phase-shifting rather than free-running as would occur under forced desynchrony protocols^14,16^. The 20 h component of locomotor activity (19.9 h) had a smaller variance (standard deviation = 0.3; equality of variance test; *P* = 0.055, vs. the 24 h component of activity) suggesting that activity is more influenced by the masking of the 10:10 LD cycle. The amplitude of the 24 h ‘circadian’ oscillation in body temperature was higher (though not statistically significant) than the 20 h body temperature component (5939.21 ± 1783.15 vs. 1347.34 ± 380.74, the 20 h component of body temperature; *P* = 0.062, paired *t*-test).

### Duration and temporal distribution of waking, NREM sleep and REM sleep in the T20 protocol

**Figure 2:**
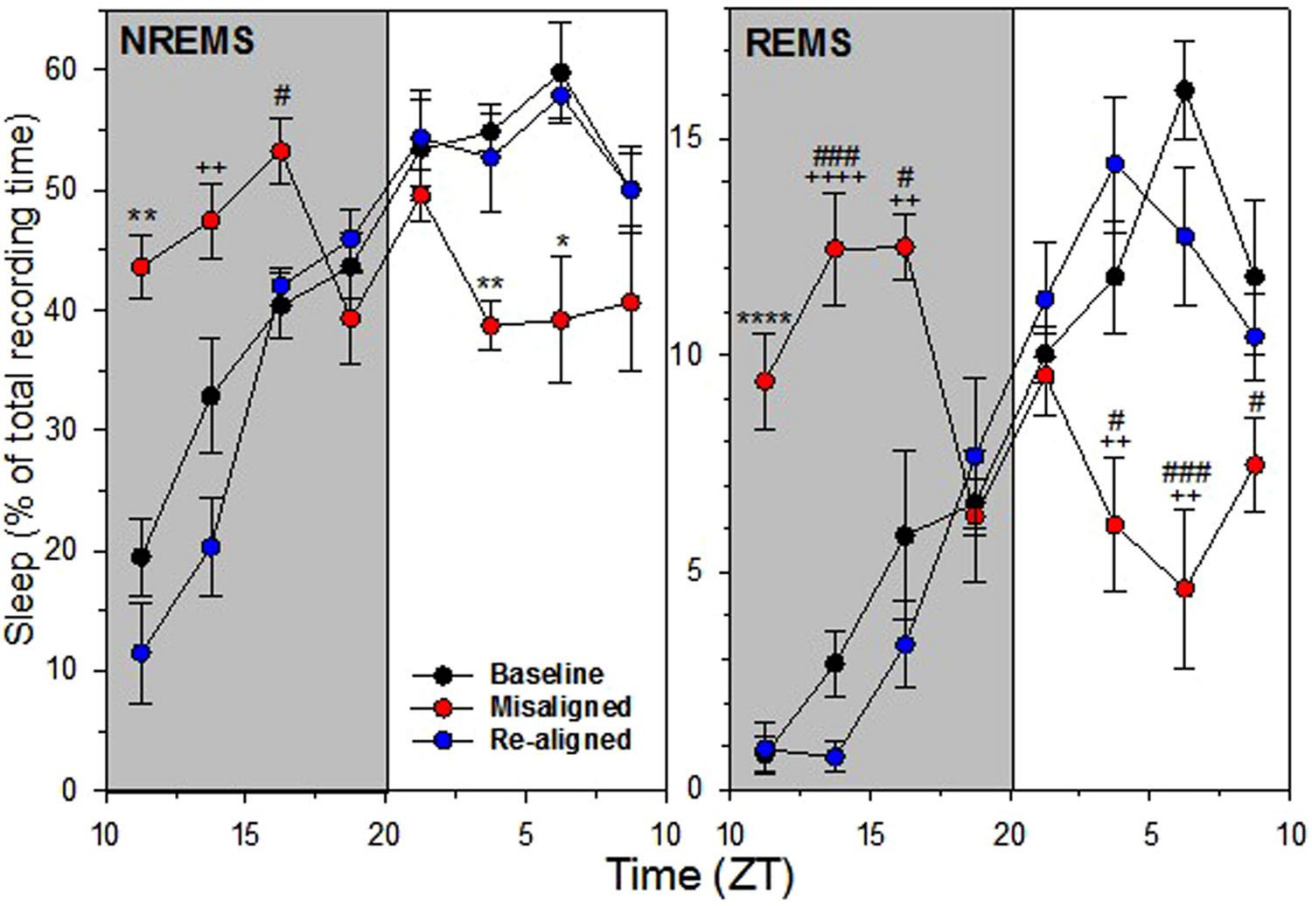
Time course of 2.5 hourly binned values of non-rapid eye movement (NREM) sleep and rapid eye movement (REM) sleep for the baseline T20 cycle (black circle symbols), misaligned T20 (red symbols) and re-aligned T20 cycle (blue symbols), expressed in percentage of recording time per 2.5 h (AVG ± SEM; B6: *n* = 5). Two and half hourly bins showing a significant effect of T20 cycle (repeated measures ANOVA, *P* < 0.05) were followed by the TUKEY *post-hoc* tests (between the three T20 cycles): Asterisks (*P < 0.05; **P < 0.01; ****P < 0.0001) indicate that the misaligned cycle is different from both, baseline and re-aligned T20 cycles; plus symbols (^++^*P* < 0.01; ^++++^*P* < 0.0001) display that the misaligned T20 cycle is different from the re-aligned T20 cycle only; and # symbols (^#^*P* < 0.05; ^###^*P* < 0.001) show that the misaligned T20 cycle is different from the baseline T20 cycle only.

The amount and distribution of vigilance states were assessed on the 1^st^ T20 cycle (baseline/aligned), 4^th^ T20 cycle (misaligned) and 7^th^ T20 cycle (re-aligned) (Fig. 1A). Whilst slight differences in re-alignment may occur between animals based upon the ~24 h period observed in each animal, cycle 7 was the closest cycle to baseline conditions in all cases. The total time spent in wakefulness, NREM sleep or REM sleep did not differ significantly between the three conditions (1-way repeated ANOVA, factor “T20 cycle”; F_2,12_ = 2.33, *P* ≥ 0.14; Table 1). To examine how the T20 cycles affect the light-dark distribution of the vigilance states, a “T20 cycle” (1^st^, 4^th^, or 7^th^) × “Phase” (Light or Dark) 2-way repeated ANOVA conducted on each vigilance states revealed a significant interaction (Wakefulness: F_2,12_ = 8.08, *P* = 0.006; F_2,12_ = 7.72, NREM sleep: *P* = 0.007; REM sleep: F_2,12_ = 7.16, *P* = 0.009). T20 cycles had a pronounced effect on vigilance states when the light and dark phases were analysed separately (1-way repeated ANOVA, factor “T20 cycle” per phase; F_2,12_ = 8.28; P ≤ 0.0055; Table 1). On the 4^th^ day of the T20 protocol the typical light-dark differences of wakefulness and NREM sleep were abolished (paired t-test, *P* = 0.13 and *P* = 0.26, respectively; Table 1). This LD ratio provides an index of nocturnality (when Sleep L/D ratio is >1) or diurnality (<1). Rhythms in REM sleep were also affected during the misaligned cycle (0.69 ± 0.12 (SEM), *P* = 0.058) when compared to the baseline cycle (3.40 ± 0.55, *P* = 0.002) and re-aligned cycle (6.27 ± 2.57.54, *P* = 0.01). Unlike NREM sleep, REM sleep appeared to be reversed under the misaligned condition, suggesting a ~24 h free-running component of REM sleep under T20 cycles as found previously under T22 cycles in rats^11^. The time course of 2.5 hourly binned values of NREM sleep and REM sleep for the fourth T20 cycle reflected this loss of nocturnality with most intervals being different from baseline except during the dark-to-light transition (Fig. 2). In conclusion, both NREM and REM sleep were dramatically affected during the misaligned cycle, and these effects were no longer observed under realigned conditions.

**Table 1:** Vigilance states under T20 conditions (% [mean ± SEM]).

		20-h	10-h Dark	10-h Light	LD Ratios	*P* (LD)
W	1^st^ T20	47.5 ± 1.4	61.9 ± 1.5	33.0 ± 2.4	0.54 ± 0.04	*0.002*
	4^th^ T20	47.5 ± 1.4	43.9 ± 2.1	51.1 ± 2.6	1.17 ± 0.09	*0.13*
	7^th^ T20	50.5 ± 0.4	66.9 ± 2.1	34.1 ± 1.8	0.51 ± 0.04	*0.001*
	*P* (T20)	*0.14*	*<0.0001*	*0.0004*		*0.006*
NREMS	1^st^ T20	44.3 ± 1.4	34.1 ± 1.4	54.5 ± 2.3	1.61 ± 0.09	*0.001*
	4^th^ T20	44.0 ± 1.0	45.9 ± 1.8	42.0 ± 1.8	0.92 ± 0.06	*0.26*
	7^th^ T20	41.8 ± 1.1	29.9 ± 1.7	53.7 ± 2.6	1.83 ± 0.17	*0.003*
	*P* (T20)	*0.27*	*<0.0001*	*0.0055*		*0.007*
REMS	1^st^ T20	8.2 ± 0.5	4.0 ± 0.6	12.4 ± 0.7	3.40 ± 0.55	*0.002*
	4^th^ T20	8.5 ± 0.7	10.2 ± 0.9	6.9 ± 1.0	0.69 ± 0.12	*0.058*
	7^th^ T20	7.7 ± 0.9	3.2 ± 0.8	12.2 ± 1.2	6.27 ± 2.54	*0.01*
	*P* (T20)	*0.14*	*<0.0001*	*0.0003*		*0.009*

Percentage of wakefulness, non-rapid eye movement sleep (NREMS) and rapid eye movement sleep (REMS) (% of total recording time), and Light/Dark (L/D) ratios (amount of vigilance state spent in the 10-h light phase/10-h dark phase) (B6: *n* = 5). Statistics are indicated by *P* values for the ‘T20 cycle effect’ (1-way ANOVA), interaction ‘T20 cycle x Phase’ (2-way ANOVA), and for the Light-Dark difference (paired *t-*tests).

**Figure 3:**
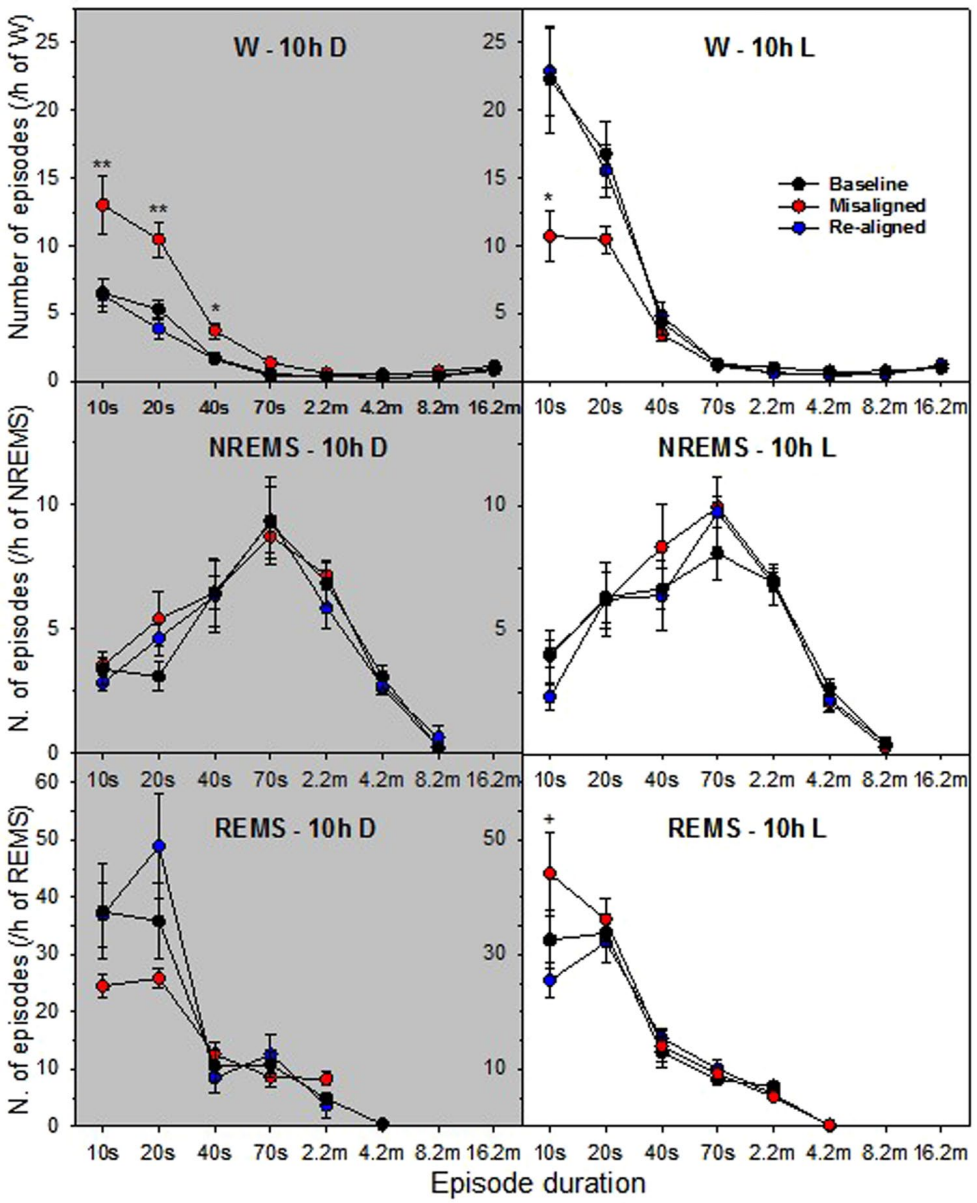
Frequency distribution of episodes of wakefulness (W), NREM sleep (NREMS) and REM sleep (REMS) throughout eight consecutive time bins (10, 20–30, 40–60, 70–120, 130–240, 250–480, 490–960 and ≥970 s) for the 10-h light and 10-h dark periods of the T20 cycles. Values are potted against the lower limit of each bin. Circle symbols (same colour codes as Fig. 2) represent the mean number of W, NREM sleep and REM sleep episodes (±SEM; B6: *n* = 5) per bin expressed per hour of its respective vigilance state (W, NREMS and REMS). Time bin with a significant effect of T20 cycle (repeated measures ANOVA, *P* < 0.05) were followed by Tukey *post-hoc* tests (between the three T20 cycles): Asterisks (*P < 0.05; **P < 0.01) indicate that the misaligned T20 cycle is different from both baseline and re-aligned T20 cycles; plus symbol (^+^P < 0.05), misaligned T20 cycle higher than re-aligned T20 cycle.

We next hypothesized that sleep or waking occurring at an inappropriate time of day would be associated increased fragmentation. Differences in the absolute amount of each vigilance state across the conditions (Fig. 2) affected this distribution (absolute data shown in Figure S1). As such, data were normalised per hour of each state (Fig. 3). Consistent with the hypothesis of increased fragmentation, during the misaligned cycle there was a relative predominance of short episodes of wakefulness (W) lasting <1 min during the 10 h dark period (Fig. 3). Mice showed a relative increase in short duration waking episodes (<30 s) during the dark phase (compared to either baseline and re-aligned cycles) and a relative reduction of waking episodes <10 s during the light phase. The reduction in very short waking episodes observed during the light phase reflects consolidated sleep and wakefulness (ANOVA, F_2,12_ = 6.88, *P* = 0.01; Tukey adjusted *post-hoc* tests: *P* < 0.05). Whilst there was a trend for higher incidence of NREM sleep episodes with a duration of 20–30 s during the dark period, this was not statistically significant (ANOVA, F_2,12_ = 2.90, *P* = 0.094; Tukey adjusted *post-hoc* test [misaligned vs. baseline/aligned cycle]: *P* = 0.08). Short REM sleep episodes of 10 s were also relatively increased during the light phase (ANOVA, F_2,12_ = 7.13, *P* < 0.01; Tukey adjusted *post-hoc* tests [misaligned vs. re-aligned cycle]: *P* < 0.05). This could suggest an increased REM sleep pressure, resulting in a higher number of ‘attempts’ to enter REM sleep. Concurrently, there may be a competing drive to initiate NREM sleep, which prevents the expression of prolonged REM sleep episodes^34^.

### Effects of T20 cycle on EEG spectra

**Figure 4:**
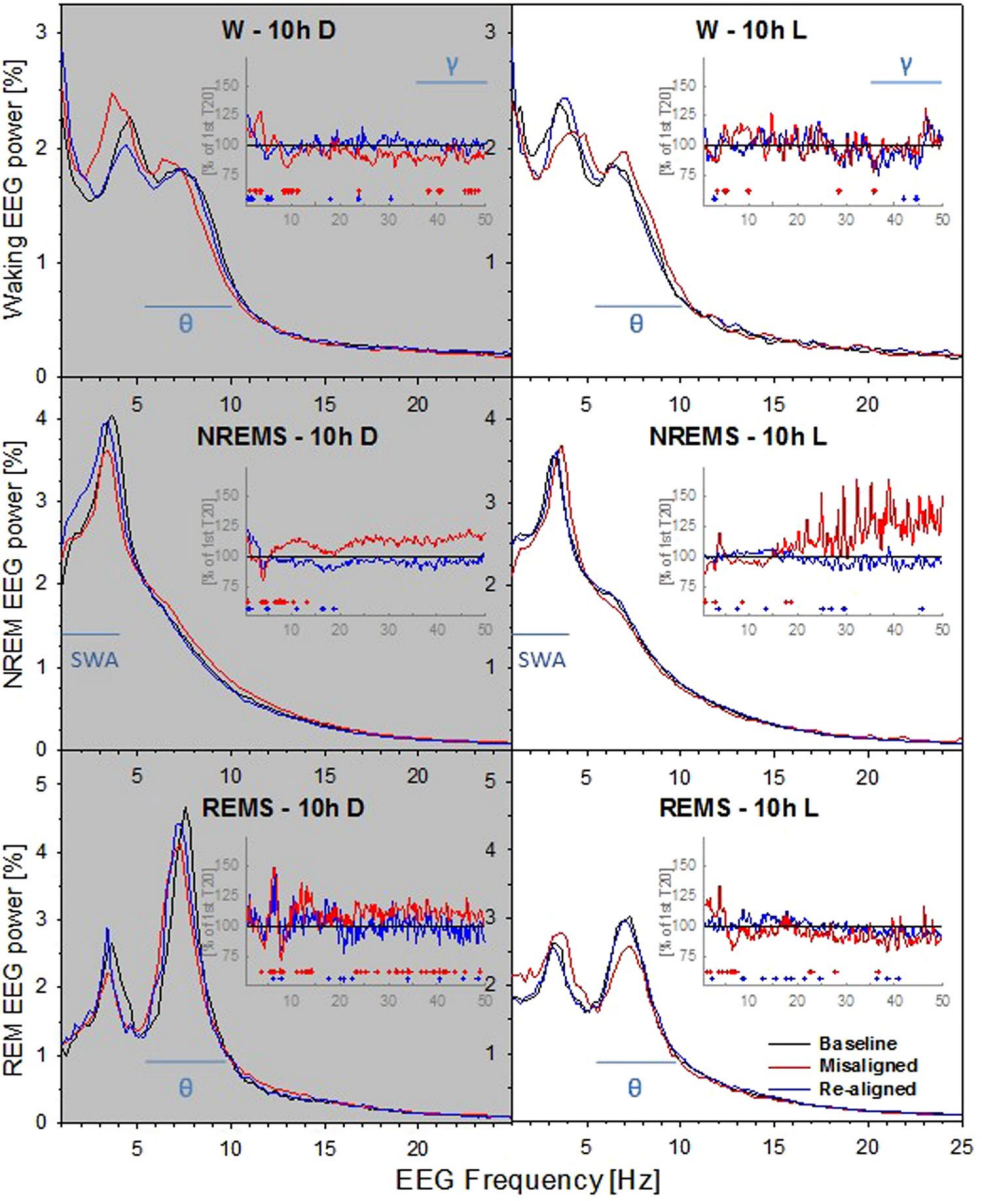
EEG spectral profiles under different T20 conditions during the 10-h dark (D - *left*) and 10-h light (L - *right*) phases, and averaged for all 10-s epochs scored as wakefulness (W - *top*), NREM sleep (NREMS - *middle*), or REM sleep (REMS - *bottom*). Average EEG spectra are normalized to total EEG power [%] over the entire 10-h dark (*left*), or 10-h light (*right*) phase. Note that for optimal viewing of the lower frequencies (<10 Hz) such as theta dynamic, the hertz scale (x-axis) in the main graphs is only displayed up to 25 Hz (instead of 50 Hz). The inset graphs illustrate the spectral differences as percent change for the misaligned (red line) and re-aligned (blue line) T20 cycles versus the baseline T20 cycle (=100%; B6: *n* = 5). Significant T20 differences are indicated by red (misaligned vs. baseline T20) and blue (re-aligned vs. baseline T20) crosshairs (*P* < 0.05, paired *t-*tests) at the bottom of each inset. Some specific EEG bands are denoted by the horizontal blue lines. SWA (slow wave activity: 1–4 Hz), θ (theta: 5.5–10 Hz) and γ (gamma: 35–50 Hz).

EEG spectral analysis revealed pronounced effects of the T20 protocol on brain activity during both the dark and light phases (Fig. 4). The EEG spectra were analysed and compared against baseline to identify the key EEG differences that were further investigated in the following section. First, we found that compared to baseline, waking EEG spectral power during the dark period of misalignment was reduced in the higher theta frequency range between 8.3 to 10 Hz as well as bins 10.5, 11 and 11.5 Hz, Fig. 4 – top/left panel. This reflects a shift of theta peak toward slower frequencies (characteristic of quiet wakefulness, see below). An opposite trend was apparent during the light period (Fig. 4, top/right panel). Consistently, EEG power within the gamma frequency range (35–50 Hz) was also reduced during the dark period of the misaligned cycle. Overall, the light-dark differences in waking EEG spectra appeared to be reversed under the misaligned cycle.

During the dark period, NREM sleep mean EEG power between 1 to 1.5 Hz was higher during the re-aligned T20 cycle. In addition, REM sleep EEG power in the theta frequency range (7.6 to 8.3 Hz) was significantly lower during the dark period of the misaligned T20 cycle (Fig. 4 – bottom/left panel) and higher in frequencies between 5.6–6.1 and bin 6.6, suggesting a shift of theta peak toward slower frequencies. In contrast to wakefulness (see above), REM sleep EEG power within the gamma frequency range (35–50 Hz) was increased during the dark period of the misaligned cycle. During the light period, relative REM sleep EEG theta power was reduced under the misaligned T20 cycle as compared to baseline (significant for bins 5.6, 6.1 and 6.6–7.3 Hz, inset graphs of bottom/right panel).

**Figure 5:**
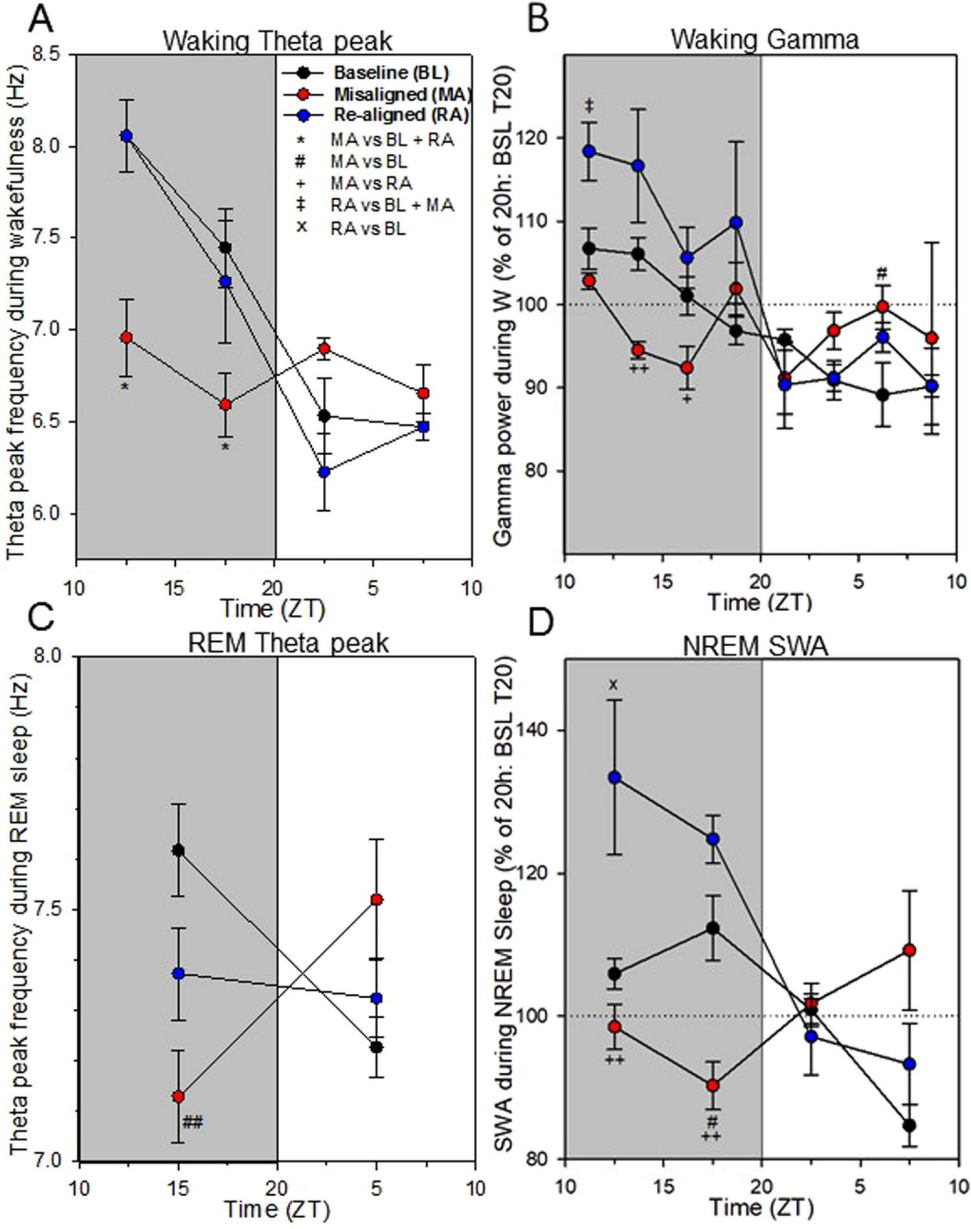
Time course analyses of EEG power density and theta-peak frequency (TPF) during vigilance states. (**A**) Time course of TPF (AVG ± SEM) during wakefulness for the baseline, misaligned and re-aligned T20 cycles. (**B**) Time course of EEG gamma power during wakefulness for the baseline, misaligned and re-aligned T20 cycles. (**C**) Time course of TPF (AVG ± SEM) during REM sleep for the 3 T20 cycles. (**D**) Time course of EEG slow wave activity during NREM sleep for the 3 T20 cycles. EEG slow wave activity (mean ± SEM) was expressed relative to the 20 hours of the baseline T20 cycle (=100%). Intervals of EEG spectral power and TPF with significant effect of T20 cycle (repeated measures ANOVA, *P* < 0.05) were followed by the Tukey *post-hoc* tests (between the 3 T20 cycles): Asterisks (*P < 0.05) indicate that the misaligned T20 cycle is different from both baseline and re-aligned T20 cycles; # symbols (^#^*P* < 0.05; ^##^*P* < 0.01), misaligned T20 cycle is different from the baseline T20 cycle; plus symbols (^+^*P* < 0.05; ^++^*P* < 0.01), misaligned T20 cycle is different from the re-aligned T20 cycle; ‡ symbol (^‡^*P* < 0.05) indicates that the re-aligned T20 cycle is different from both baseline and misaligned T20 cycles; and X symbol (^X^*P* < 0.05) indicates that the re-aligned T20 cycle is different from the baseline T20 cycle only.

As suggested above, the time course analysis of theta peak frequency (TPF) during wakefulness confirmed a significantly lower TPF over the dark period of the misaligned cycle (>1 Hz reduction during the first half; ~ 0.75 Hz reduction for the 2^nd^ half of the 10-h dark period; Tukey adjusted *post-hoc* tests: *P* < 0.05, Fig. 5A). However, an opposite profile and a trend for faster TPF was observed during the light period of the misaligned cycle (0.7 Hz increase for the 1^st^ half of the 10-h light period, compared to the re-aligned cycle; 6.9 vs. 6.2 Hz; adjusted *post-hoc* test: *P* = 0.064). In addition, time course analysis of gamma frequency power during wakefulness revealed that it was reduced in the middle of the dark period during the misaligned cycle, as compared to the baseline and re-aligned cycles (Fig. 5B), and a higher gamma power in the middle of the light period (3^rd^ light interval: misaligned vs. baseline; adjusted *post-hoc* test: *P* < 0.05) was found. Furthermore, the daily variation in gamma power was no longer apparent during the misaligned cycle (“Time” 1-way repeated ANOVA, F_7,28_ = 1.20, *P* = 0.33) in comparison to the baseline and re-aligned days (“Time” 1-way ANOVAs, F_7,28_ = 5.99 and 6.10, *P* = 0.0002). After further analysis of REM sleep TPF during the misaligned cycle, this theta frequency shift was indeed confirmed with a significantly slower theta peak by 0.5 hertz compared to baseline TPF (shift from 7.617 to 7.129 Hz; Tukey adjusted *post-hoc* tests: *P* < 0.01, Fig. 5C).

To further investigate whether misaligned circadian rhythms affect sleep regulation, the time course of EEG slow wave activity (1–4 Hz) during NREM sleep (an indicator of sleep pressure and sleep intensity) was calculated. EEG slow wave activity in NREM sleep typically increases during the dark periods after mice spend extended periods awake, and declines during the light periods, when NREM sleep is prevalent. These usual trends were present across the baseline and re-aligned cycles but were reversed under the misaligned cycle (Fig. 5D). Moreover, NREM sleep slow wave activity during the dark period of the re-aligned T20 cycle was also higher compared to the baseline T20 cycle (significant in the first half of the dark period [ZT10-15]; ANOVA, F_2,12_ = 9.36 P < 0.01; Tukey adjusted *post-hoc* test [re-aligned vs. baseline cycle]: P < 0.05).

### Effects of individual tau on re-alignment

**Figure 6:**
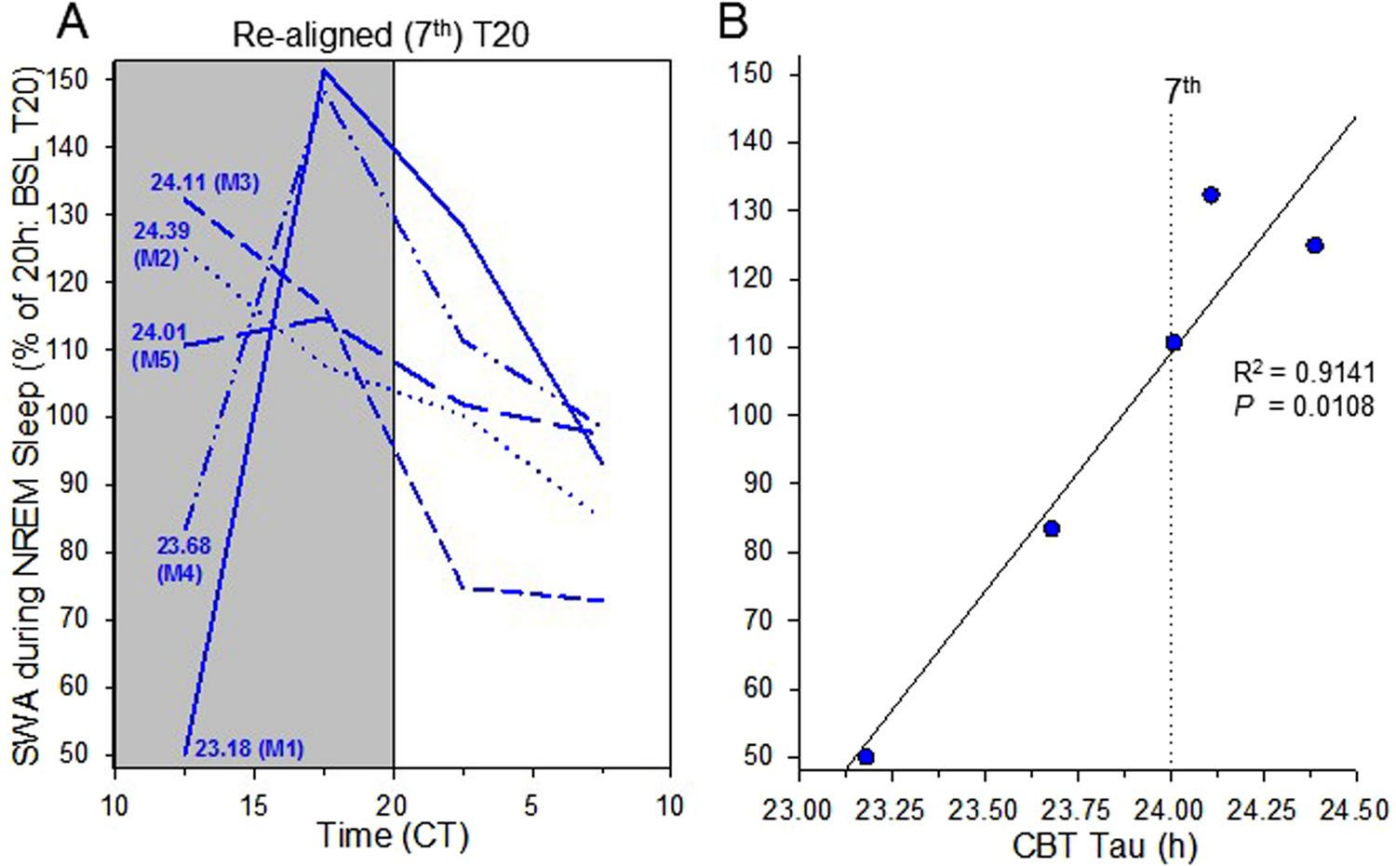
Effects of individual period on NREM sleep slow wave activity (SWA). (**A**): Individual time course of corrected EEG SWA based upon the individual animal’s CBT period (for the re-aligned T20 cycle). The numbers at the first SWA interval indicate the individual animal’s period (for the 5 mice M1 to M5). Individual EEG SWA was expressed relative to the 20 hours of the baseline T20 cycle (=100%). (**B**): Correlations between NREM SWA during the first “re-aligned” interval with the individual animal’s CBT period. Pearson’s correlation coefficient (R-squared) and its *P* value are indicated on the graph.

The effects of NREM sleep slow wave activity during the re-aligned cycle could be explained by the effects of the preceding circadian misalignment on subsequent sleep. Alternatively, this “re-aligned” state may more accurately be defined as a state with potential varying degree of circadian misalignment between individual animals. To investigate this further, NREM sleep slow wave activity was analysed in animals based upon their individual period. As locomotor activity is acutely suppressed by light (negative masking), period was derived from core body temperature (Fig. 1B). When NREM sleep slow wave activity was plotted in this manner, it was immediately evident that animals with a period of <24 h show a much lower slow wave activity, whereas mice with a period >24 h show a higher slow wave activity (Fig. 6A). This variability in individuals results in a high variance in the first half of the dark (active) period in the 7^th^ 20 h cycle, which we refer to as “re-aligned”. Indeed, a strong positive correlation exists between NREM sleep slow wave activity and the period of core body temperature (Fig. 6B, R^2^ = 0.914, P = 0.011).

## Discussion

Here we characterise the effects of T-cycles on the timing and distribution of waking and sleep, as well the spectral composition of the EEG signal over the course of 10 days. We show that T20 cycles influence the distribution of vigilance states as well as EEG slow wave activity, and theta and gamma frequencies. Most of these differences were specific to the cycle of testing, and the effects on timing and distribution of vigilance states were most pronounced when internal circadian time was misaligned with respect to the external LD cycle. By contrast, these effects were minimal when animals reached a cycle in which internal circadian time coincided with external LD cycle. Here we show for the first time that even though NREM and REM sleep profiles appear normal when animals were approximately re-aligned, changes in the EEG spectra persisted. Perhaps the most important of these changes is an elevation of slow wave activity in NREM sleep, indicative of increased homeostatic sleep pressure. We go on to show that these effects are influenced by the ~24 h period expressed by individual animals, with a strong positive correlation between NREM slow wave activity and the period of core body temperature. These findings confirm and extend the findings of Rozov *et al*.^33^. Such changes are typically observed following sleep deprivation and suggest that even when the amount and distribution of sleep appear normal following T-cycles, these protocols may result in non-restorative sleep with consequences for subsequent behaviour.

Previous EEG studies in the rat have shown that wakefulness and NREM sleep show two stable rhythms under a T22 cycle, corresponding to the period of the external LD cycle (22 h) and the internal circadian clock (24 h). By contrast, REM sleep displayed only one robust 24 h rhythm, indicative of circadian regulation alone^11^. Here we show that the LD ratio in REM sleep was reversed during the misaligned cycle with increased REM sleep during the dark phase (0.69 ± 0.12, compared to the baseline cycle 3.40 ± 0.55). These findings also suggest that REM sleep is also primarily regulated by the 24 h period of the circadian clock rather than the 20 h period of the LD cycle (Fig. 2). REM sleep fragmentation similarly appears reversed during the misaligned cycle (Fig. 3). These data suggest that under such T-cycles, REM sleep free-runs and is unaffected by the period of the external LD cycle. These data support human studies, which show that REM sleep is under strong circadian modulation^22^, particularly when NREM sleep ‘pressure’ is low^35^. As has been shown previously, the fragmentation of sleep and wakefulness is influenced by circadian time and preceding sleep-wake history^36,37^. However, the relative fragmentation of wakefulness is strongly affected under the T20 protocol, as the normal light-dark difference of fragmentation distributions was not reversed but abolished. This may be similar to an arrhythmic animal^38^.

The wakefulness EEG theta peak frequency (TPF, Fig. 5A) was slower during the dark phase of the misaligned T20 cycle - comparable to that observed in the light phase during baseline and re-aligned T20 cycles when quiet wakefulness is prominent. Moreover, waking TPF was not significantly higher during the first half of the light phase during the misaligned cycle, and was not comparable to dark phase levels during baseline and re-aligned T20 cycles when active wakefulness is dominant. In summary, these data show that the normal LD differences in waking TPF seen under baseline and re-aligned conditions are abolished during the misaligned cycle. The recent data of Rozov *et al*.^33^ also showed that the circadian regulation of waking theta power gradually disappeared under T22 and T21 cycles.

In our study, waking gamma (35–50 Hz) power was affected by T-cycles (Fig. 5B). Indeed, the circadian regulation of waking gamma power gradually disappeared under T22 and T21 cycles^33^. Since waking EEG gamma power is typically an indicator of attentive wakefulness in rats^39^ and is also induced by wake-promoting drugs in mice^40,41^, we hypothesize that circadian misalignment may target brain systems implicated in higher cognitive functions, such as attention or learning. Consistent with this hypothesis, a recent study in mice has shown that a reduction of cortical gamma oscillation impairs performance in a long-term memory version of the novel object recognition (NOR) test, but neither in the Y maze (short-term spatial working memory) nor in fear-conditioning tests^42^.

Notably, REM sleep spectral EEG power was also affected during circadian misalignment. Specifically, during the light phase of the circadian disruption T20 cycle, EEG theta power during REM sleep was reduced compared to the baseline and re-entrained T20 cycles. Moreover, during the dark phase of the misaligned T20 cycle, the REM sleep theta peak frequency was shifted toward slower frequencies compared against dark baseline T20 cycle (Fig. 5C). This theta-peak shift could be related to a ~1 °C reduction of cortical temperature^43^, since core body temperature remained overall lower during the dark period (vs light period) of the misaligned cycle. These REM sleep spectral changes were still evident during the dark phase of the re-aligned T20 cycle, when circadian time and the external LD cycle were again in phase. Consistently, human studies using forced desynchrony protocols have shown that REM-specific EEG power (alpha activity: 8.25–10.5 Hz) becomes desynchronised from core body temperature^44^. This is also consistent with the constant ‘sleep pressure protocol’ in rats, which revealed that activity in the 3–7 Hz frequencies in the REM sleep EEG are under strong circadian regulation^32^.

NREM sleep EEG slow wave activity is dependent on the preceding sleep history, with less influence of the circadian process^22,45^. However, strong circadian regulation of EEG slow waves has recently been shown in humans, as manifested in a significant time of day effect on several slow wave parameters such as their incidence, amplitude, and slope^46^. The peaks of the circadian rhythms in these slow wave parameters were situated during the biological day. In our study, across the baseline and re-entrained T20 cycles, NREM sleep slow wave activity showed a sleep-dependent modulation and a smaller circadian modulation (Fig. 5D). Surprisingly, mice had a higher slow wave activity during the re-aligned T20 cycle, when internal circadian time and the external environment were again aligned. As we describe above, T20 cycles have clear effects on the phasing and relative fragmentation of sleep, but these circadian effects were not observed when re-aligned. As such, the increased slow wave activity observed even when sleep appears normal may reflect an accumulation of sleep debt after poor sleep quality as a result of the T20 paradigm. Poor sleep quality could be defined here by changes in sleep parameters (e.g., trend of reduced total sleep, LD ratios, relative fragmentation of vigilance states) as a result of sleeping during circadian misalignment. Many clock gene transgenic mouse models including *Cry1/2*^−/−^, *Bmal1*^−/−^ and *Per3*^−/−^ also show higher slow wave activity when compared to their respective wild-type controls^37,47,48^. Whilst in this current study we house animals under non-24h LD cycles, housing transgenic animals with a non-24h circadian clock under 24 h LD cycles may similarly be expected to have consequences for sleep. Finally, the effects of T20 cycles on slow wave activity could also potentially arise from a pleiotropic role of circadian genes in sleep homeostasis^49,50^.

A alternative explanation is that the “re-aligned” state we describe may more accurately be defined as a state with potential varying degree of circadian misalignment between individual animals. To test this, NREM sleep slow wave activity was analysed based upon the individual animal’s period, determined from core body temperature. This shows that the individual’s period is highly predictive of the level of NREM slow wave activity during this “re-aligned” state, with animals with period <24 h showing low NREM slow wave activity, whereas animals with period >24 h show increased NREM slow wave activity (Fig. 6). As animals with a circadian period <24 h would normally be active before the onset of the dark period, the prevailing light cycle at this point may result in an extended rest phase. By contrast, animals with circadian period >24 h would not normally start their activity onset until later in the dark period, resulting in a shorter preceding rest phase. Differences in phasing between an animal’s core body temperature and the normal light (rest) phase may also influence preceding sleep. These findings emphasise that the relationship between internal biological time and the external environment in regulating sleep.

T-cycles have been used to study the effect of circadian disruption on a number of different physiological and behavioural parameters^12,13^. In such studies, animals are often housed for long periods under these conditions (8–10 weeks). Our data show that even 7 days under T20 cycles produce variable effects on sleep, even after NREM and REM sleep profiles return to baseline levels. It remains to be established whether increased slow wave activity following T-cycle exposure is comparable to that seen following acute sleep deprivation. It is well established that sleep deprivation and sleep restriction have effects on both mood^26,51^ and cognition^27,52–57^ in humans and rodents. It is unclear whether the cognitive effects of circadian disruption are a primary consequence of desynchrony between circadian clocks in the brain and the external environment, or result from changes in sleep^58^. Cognitive and behavioural deficits described in circadian knockout or mutant mice^59–62^ may also be related to sleep disturbances that occur in these animals^23^. Thus, changes in sleep may reflect a key mechanism underlying changes in cognitive performance and emotional processes as a result of circadian disruption. Our data suggest that circadian disruption may have subtle and long-lasting effects on sleep, which may provide a mechanism to explain why these conditions give rise to adverse effects on health.

## Materials and Methods

### Animals

Sample size calculations were based upon an effect size of 1.5, with alpha = 0.05 and power = 0.80. Whilst this would require a sample size of n = 8 to detect a difference between two independent samples, based upon the same parameters, a within-groups design would obtain the same power with n = 5. All calculations were performed using G*power^63^. Five C57BL/6J (B6) male mice were purchased from Envigo (Bicester, UK) at the age of 11–13 weeks. Animals were singly-housed, and were provided with food and water ad libitum. They were kept in a temperature-controlled (21–22 °C), light-tight and sound attenuated cabinet with a 12-hr:12-hr light:dark (LD) cycle (T24) with light provided by cool white LEDs with an irradiance (during the light period) of 50 lux at the cage bottom. Sizzle nest bedding was provided to aid thermoregulation. All experimental procedures were approved by the University of Oxford Clinical Medicine Animal Welfare and Ethical Review Board and were carried out under U.K. Home Office Project Licence 30/2812 and personal licence I459D3D59. All experimental methods were performed in accordance with the veterinary guidelines and the University of Oxford policy on the use of animals in scientific research. Animals were housed in specific pathogen free (SPF) conditions, and the only reported positives on health screening over the entire time course of these studies were for *Helicobacter hepaticus* and *Entamoeba sp* (Envigo, Alconbury UK).

### Surgery

A telemetric transmitter (volume, 1.9 cm^3^; total weight, 3.9 g; TL11M2-F20-EET; DSI, St. Paul, MN, USA) connected to electrodes for EEG and electromyography (EMG) recordings was implanted in each animal at the age of 15.9 ± 0.5 [mean ± sem] weeks old, as previously described (Hasan *et al*., 2014). Briefly, under isoflurane anaesthesia (induction 4.5%, maintenance 0.7–2.25%), two stainless-steel EEG electrodes (length of screw shaft, 2.4 mm; outer diameter of screw thread, 1.19 mm) were implanted epidurally over the right frontal (1.7 mm lateral to midline, 1.5 mm anterior to bregma) and parietal (parietal: 1.7 mm lateral to midline, 1.0 mm anterior to lambda) cortices (Franken *et al*., 1998) and connected to the telemetry transmitter via medical-grade stainless-steel wires (surrounded by silicone tubing). The EEG electrodes and connections to the subcutaneous wiring were covered with dental cement (RelyX Arc; Kent Express, Kent, UK). Two EMG stainless-steel leads were inserted into the neck muscle ~5 mm apart and sutured in place. The telemetry transmitter was placed into the abdominal cavity of the mouse (‘intraperitoneally’). The transmitter contains one Negative Temperature Coefficient (NTC) thermistor with an operating range of 34 to 41 degrees Celsius (resolution of 0.05 degree C). A ‘PhysioTel’ receiver (RPC-1; DSI) underneath the cage provides reception of the telemetered data. This receiver is capable of detecting movements (activity) by sensing changes in signal strength as the animal traverses the cage. All procedures were conducted using aseptic technique. Perioperative analgesics were administrated at the onset of surgery as well as postoperatively (buprenorphine, 0.1 mg/kg; meloxicam, 5 mg/kg), as well as the following day (meloxicam, 5 mg/kg). Saline (0.9%, 500 µl) was also administered by subcutaneous injection at the end of the surgery.

### T20 cycle protocol

After a surgery recovery of 6.4 ± 0.8 (mean ± sem) weeks (under normal 24-h [12:12] LD cycles), the animals were subjected to a 20-h LD cycle, with 10 h of light and 10 h of dark. Subsequently, continuous EEG/EMG, body temperature and gross motor activity recordings were obtained under a series of twelve 10-hr:10-hr light:dark (LD) cycles (T20). The baseline and re-entrained 10:10 LD cycle started at dark-onset (ZT12 of the previous 12:12LD, Fig. 1). Periodogram analyses of locomotor activity and core body temperature were performed from the start of the 2^nd^ T20 until the end of the 11^th^ T20 cycle. Note that the baseline (1^st^) and 12^th^ T20 cycles were excluded from the periodogram analyses as these cycles were mainly on the same phase of the preceding T24.

### EEG-assessed vigilance states and spectral analyses

The EEG and EMG signals were conditioned with a one pole high-pass (−1.1 dB at 1.0 Hz; −3.8 dB at 0.5 Hz) and a two pole low-pass antialiasing (−1.6 dB at 50 Hz) analogue filters built in the transmitter. The telemetric EEG and EMG data were transmitted at 455 kHz to an RPC-1 receiver (DSI) and sampled at 250 Hz. An additional (30th order low-pass FIR) digital filter was selected at 49.93 Hz (2 dB of attenuation). Detailed analyses of vigilance states and the EEG were performed on the data recorded during the 1^st^ (baseline) day of T20 cycle, 4^th^ day of T20 (misaligned), and 7^th^ day of the protocol (realigned). Vigilance states were annotated based on 10-sec epochs by visual inspection of the EEG and EMG signal simultaneously displayed on a PC screen, according to standard criteria of 4-sec epochs scoring^64^. The time spent in waking, NREM sleep and REM sleep was expressed as a percentage of total recording time over time intervals of 2.5 hours. To investigate the effects of T20 protocol on sleep/wake architecture, consolidated uninterrupted episodes of wakefulness, NREM sleep and REM sleep were detected, and grouped as a function of their duration (10, 20–30, 40–60, 70–120, 130–240, 250–480, 490–960 and ≥970 sec) as previously^36^). Note that brief awakenings immediately preceded and followed by consolidated sleep and lasting less than 5 sec were not considered an interruption of sleep. However, if >50% of a 10-sec epoch was characterised by increased EMG and disappearance of slow waves, the entire 10 sec epoch was annotated as wakefulness. EEG power spectra were computed for consecutive 10-s epochs by a fast Fourier transform (FFT; frequency range, 0.977–49.805 Hz; resolution, 0.244 Hz; Hanning window function). Epochs contaminated by artefacts were excluded from EEG spectral analyses (% of total recording time: baseline = 6.14 ± 0.94%; misalignment = 6.86 ± 1.89%; and realignment = 5.41 ± 1.07%). EEG power spectra were calculated for the three vigilance states during the 10-h dark and 10-h light periods, and analysed using several complementary approaches as follows. First, to assess the relative contribution of specific frequencies in the EEG signal, EEG power spectra during the light and the dark phase were represented as a percentage of corresponding total (0.977–49.805 Hz) EEG power for each vigilance state. Second, to investigate the effect of T20 cycle, absolute EEG power in each frequency bin during the experimental conditions (misaligned cycle, realigned cycle) was expressed as a percentage of the mean power in the same bin during the first (baseline) cycle. EEG slow wave activity during NREM sleep was computed by averaging absolute EEG power in the frequency range between 0.977–3.906 Hz within each 10-sec epochs. Subsequently, to investigate the time course of SWA, the recording was subdivided into 5 hours intervals, and corresponding absolute SWA values were averaged per interval. The values of absolute SWA were then expressed as percentage of mean NREM sleep SWA calculated over the entire 20-h ‘baseline’ period of T20 protocol for each individual mouse. EEG gamma power during wakefulness was computed by averaging the absolute EEG power in the frequencies ranging from 35.156 to 49.805 Hz. Absolute mean values were calculated by dividing the 10-h phases into four 2.5 hours intervals. Absolute mean values were also individually normalized to the mean gamma power in wakefulness for the entire 20-h ‘baseline’ period on T20 cycle 1. Theta-peak frequency (TPF) in waking EEG and REM sleep was defined as the frequency bin corresponding to the maximal EEG power in the range between 5.5–10 Hz over time intervals of 5 and 10 hours, respectively. The larger time interval (10-h) permitted to have a sufficient amount of REM sleep.

### Statistical analyses

Lomb-Scargle periodogram analyses were performed on the activity/temperature data from the 2^nd^–11^th^ T20 cycle using ActogramJ^65^ running through ImageJ (1.47 v, NIH, USA), showing the presence of significant rhythms (P < 0.0001). The rest of the circadian and EEG data were analysed using SAS v9.4 (SAS Institute, Cary, NC, USA). Variance comparison (between the periods of *T 20*-h and *τ ~24*-h component) was performed with the equality of variances test (PROC TTEST). Comparisons of the powers of period peaks (between activity & body temperature; and between the powers of the 20 h and 24 h component) were conducted with paired t-test (PROC TTEST). Paired t-tests were also used to check for Light-Dark difference of vigilance states, and to contrast T20 differences (circadian disruption vs. baseline T20; and re-entrained vs. baseline T20) of EEG spectral profiles (on log-transformed values). For the rest of the EEG analyses, the Mixed procedure for repeated measures analysis of variance (ANOVA as a Mixed model) was applied, and when significant (*P* < 0.05), were followed by LSMEANS (least square means) with TUKEY adjustments for *post-hoc* analyses, when comparing each T20 cycle with the 2 other T20 cycles.

## Electronic supplementary material

Supplemental figure 1
