# Supplementary material for: Effects of circadian misalignment on sleep in mice
Source: Sci Rep. 2018 Oct 26;8:15343. doi: 10.1038/s41598-018-33480-1 (PMC6203841; doi:10.1038/s41598-018-33480-1)
Supplement: Supplementary file 1 — Supplemental figure 1 [file 41598_2018_33480_MOESM1_ESM.pdf]

Effects of circadian misalignment on sleep in mice

Sibah Hasan[1], Russell G. Foster[1], Vladyslav V. Vyazovskiy[2]* and Stuart N. Peirson[1]*

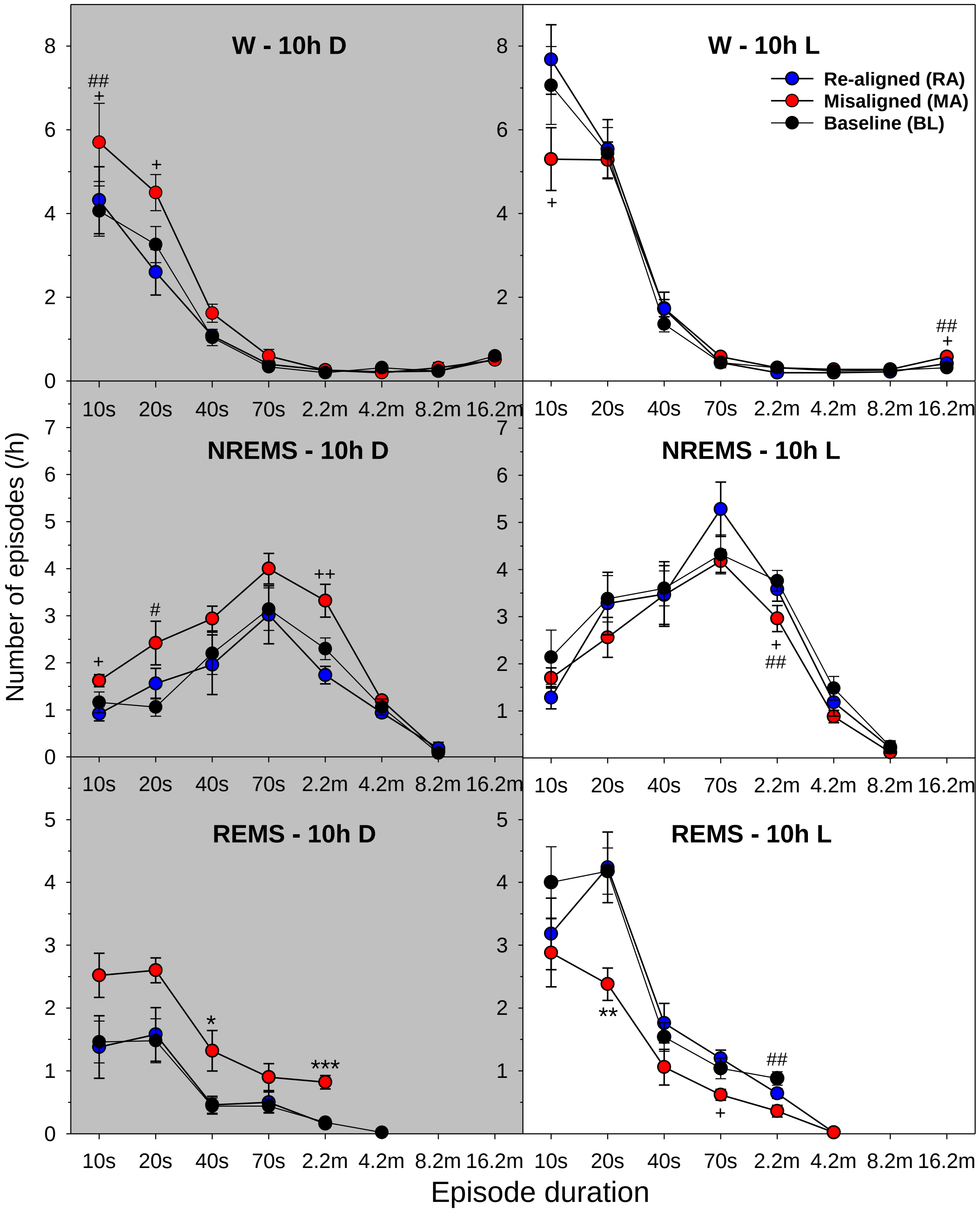

Supplemental figure 1. Frequency distribution of episodes of wakefulness (W), NREM sleep (NREMS) and REM sleep (REMS) throughout eight consecutive time bins (10, 20-30, 40-60, 70-120, 130-240, 250-480, 490-960 and ≥ 970 s) for the 10-h light and 10-h dark periods of the T20 cycles. Values are potted against the lower limit of each bin. Circle symbols (same colour codes as Fig. 2) represent the mean number of W, NREM sleep and REM sleep

episodes (± SEM; B6: *n* = 5) per bin expressed per hour of its respective vigilance state (W, NREMS and REMS). Time bin with a significant effect of T20 cycle (repeated measures ANOVA, *P* < 0.05) were followed by Tukey *post-hoc* tests (between the three T20 cycles): Asterisks (*: *P* < 0.05; ** *P* < 0.01; *** *P* < 0.001) indicate that the misaligned T20 cycle is different from both baseline and re-aligned T20 cycles; # symbols (##: *P* < 0.01), misaligned T20 cycle is different from the baseline T20 cycle; plus symbols (+: *P* < 0.05; ++: *P* < 0.01), misaligned T20 cycle is different from the re-aligned T20 cycle.
